# The Role of Autophagy in Inflammatory Bowel Disease

**DOI:** 10.3389/fphys.2021.621132

**Published:** 2021-02-03

**Authors:** Bo-Zong Shao, Yi Yao, Jun-Shan Zhai, Jian-Hua Zhu, Jin-Ping Li, Kai Wu

**Affiliations:** The 8th Medical Center of General Hospital of the Chinese People’s Liberation Army, Beijing, China

**Keywords:** inflammatory bowel disease, autophagy, inflammasome, apoptosis, mutation

## Abstract

Inflammatory bowel disease (IBD) is an idiopathic intestinal inflammatory disease, including ulcerative colitis (UC) and Crohn’s disease (CD). The abnormality of inflammatory and immune responses in the intestine contributes to the pathogenesis and progression of IBD. Autophagy is a vital catabolic process in cells. Recent studies report that autophagy is highly involved in various kinds of diseases, especially inflammation-related diseases, such as IBD. In this review, the biological characteristics of autophagy and its role in IBD will be described and discussed based on recent literature. In addition, several therapies for IBD through modulating the inflammasome and intestinal microbiota taking advantage of autophagy regulation will be introduced. We aim to bring new insight in the exploration of mechanisms for IBD and development of novel therapeutic strategies against IBD.

## Introduction

Traditionally, it is well acknowledged that the intestinal tract is the largest digesting organ. It mainly functions in absorbing food and nutrition orally taken into the intestinal lumen majorly through the absorptive enterocytes located in the gut lacunae and plica (Choi and Snider, 2019; Ruder et al., 2019; Yamamoto et al., 2020). In the recent decade, an increasing number of studies have revealed that the gut is also regarded as a critical immune organ because of its exposed and challenged with exogenous invasive pathogenic microorganisms in the gut lumen (James et al., 2020; Muller et al., 2020; Zhou et al., 2020). Several self-protective mechanisms are induced with the invasion, including the triggering of the inflammasome formation, neutrophil extracellular traps, pro-inflammatory cytokine secretion and other inflammatory, and immune responses in the gut wall. Those mechanisms largely limit the invasion of pathogenic factors through the gut wall (Bischoff et al., 2014; Tong and Tang, 2017; Ye et al., 2019; Zhou et al., 2020). However, despite of the numerous researches on the gut, there are still unsolved questions or even contradictions on the issues of the development and component of the immune system in the gut and how the intestinal immune system responds to the external and internal stress. So far, a large number of studies have revealed that during the process of intestinal self-defense system, the intestinal mucosal barrier has been widely investigated and recognized as the most important factor in the intestinal defense system. The intestinal mucosal barrier functions in maintaining the gut microbiota homeostasis and peaceful coexistence in organisms (Sanchez de Medina et al., 2014; Jarret et al., 2020). In the following contents, the components, functions, and relations with intestinal diseases will be introduced in detail.

So far, it is revealed that the intestinal mucosal barrier is generally comprised by three layers, including the mucus layer on the surface, epithelial cell layer, and inflammatory and immune cell layer in the submucosa (Zhang et al., 2017; Wang et al., 2018c; Schroeder, 2019). The mucus layer is referred to as a gel-like layer on the surface of the mucosa containing numerous kinds of proteins, carbohydrates, and lipids in mucus. The mucus layer functions in avoiding the direct contacting between epithelial cells and intestinal microbiota (Vita et al., 2019; Pelaseyed and Hansson, 2020). The intestinal epithelial cells in the second layer of intestinal mucosal barrier are mainly including absorptive enterocytes, Paneth cells, goblet cells, micro-fold villus cells, enteroendocrine cells, and tuft cells. The containing of those cells contribute to the absorption of nutrition and secreting mucus and functional proteins (Goll and van Beelen Granlund, 2015; Wu et al., 2019; Yin et al., 2019). Besides, the inflammatory and immune cells in the submucosa, such as macrophages, neutrophils, and lymphocytes, can trigger self-defensive responses through endocytosis, antigen presentation, and secretion of cytokines. Those cells in the third layer fight against the invasion of pathogens (Bui et al., 2018; Luissint et al., 2019; Ho et al., 2020). However, the disturbance of microbiota balance and damage of intestinal mucosal barrier tend to trigger the over-induction of self-defensive processes, including oxidative stress, inflammasome formation, and so on. The overwhelming induction of those self-defensive mechanisms results in several intestinal disorders such as inflammatory bowel disease (IBD; Ramos and Papadakis, 2019; Han et al., 2020; Watanabe et al., 2020). As a result, targeting on the maintaining of intestinal microbiota homeostasis and suppressing the overwhelmingly induced self-defensive inflammatory and immune responses serves as an effective pathway for the treatment of IBD.

Autophagy is regarded as a vital metabolic process. It is for degrading and recycling long-lived or misfolded proteins and useless organelles under stress conditions relying on lysosomes (Dikic and Elazar, 2018; Racanelli et al., 2018). Since initially reported in the mid-1950s, autophagy has been demonstrated to be involved in the pathogenesis and progression of various kinds of diseases (Li and Chen, 2019; Yamaguchi, 2019; Keller et al., 2020; Yim and Mizushima, 2020). In IBD, autophagy has been widely revealed to regulate the onset and development of IBD *via* immune and inflammatory modulation (Iida et al., 2017; Palmela et al., 2018; Wang et al., 2018b; Hooper et al., 2019). Modern studies indicate that targeting on autophagy might serve as a promising therapeutic strategy for the treatment of IBD. Based on those evidence, here in this paper, the latest literature on autophagy and IBD will be reviewed and the role of autophagy in IBD will be discussed. We aim to provide novel insight in the treatment of IBD taking advantage of autophagy in this review.

## Part I: Autophagy in IBD

### Biological Characteristics of Autophagy

Autophagy is a catabolic cellular process, through which some protein aggregates and damaged organelles are degraded into metabolic elements *via* lysosomes for recycling to maintain the homeostasis and vitality of cells (Allen and Baehrecke, 2020; Yang and Klionsky, 2020). The process of autophagy is widely existed in almost all kinds of cells and evolutionarily conserved from yeast to mammals (Bento et al., 2016; Reggiori and Ungermann, 2017; Tyler and Johnson, 2018). According to the mode of cargo delivery and physiological function, autophagy is mainly divided into three classic forms, including macroautophagy, microautophagy, and chaperone-mediated autophagy (Xilouri and Stefanis, 2016; Wang et al., 2018a; Cerri and Blandini, 2019). Macroautophagy is uncovered as a catabolic process featured with sequestration of degrading materials into double-membraned autophagosomes, which are subsequently fused with lysosomes for degradation (Yu et al., 2018; Lieberman and Sulzer, 2020). Microautophagy is a non-selective form of autophagy, through which cytoplasmic degrading materials are engulfed *via* invagination of the lysosomal/vacuolar membranes (Mijaljica et al., 2011; Li and Hochstrasser, 2020). Chaperone-mediated autophagy is a selective autophagy form relying on the presentation of chaperones *via* certain target motif in the substrate proteins and lysosomal chaperons (Yang et al., 2019; Dash et al., 2020). Since macroautophagy is so far the most studied form of autophagy and has been reported to be involved in diseases, here in this review, we will mainly discuss the biological features of macroautophagy and its role in IBD (herein referred to as “autophagy”).

The induction of autophagy mainly includes two steps according to our previous reviews (Shao et al., 2016; Wang et al., 2018a). In the first step, substrate materials, such as aggregated proteins, are surrounded by a cup-shaped phagophore with lipid bilayer membrane, which subsequently sequestrated into double-membrane sphere-shaped autophagosomes. The formation of phagophores demands the formation of the autophagy-related gene 1 (ATG1) complex and Class III phosphatidylinositol 3-kinase (PI3K) complex, with Unc-51-like kinase (ULK1, aka Atg1 in yeast), FIP200, ATG13, ATG101 assembly for ATG1 complex and Beclin-1, ATG14, vacuolar protein sorting 15 (VPS15), and VSP34 for PI3K complex (Bento et al., 2016; Wang et al., 2018a). The process of membrane expansion from phagophores to autophagosomes is dependent on the formation of ATG5-ATG12-ATG16L1 complex (Sheng et al., 2018). In the second step, autophagosomes dispose of “coat proteins (LC3-II)” on the surface and fuse with lysosomes with the assistance of ATG3 and ATG7 for the formation of the functional autolysosomes (Nath et al., 2014). On the occurrence of autophagy, more than 30 ATGs are involved. In addition, two classic signaling pathways are vital in the regulation of autophagy process. The Class I PI3K-mammalian target of rapamycin (mTOR) is illustrated as an inhibitory pathway of autophagy *via* the stimulation of mTOR complex 1 (mTORC1), with AMP-activated protein kinase (AMPK) considered as the classic upstream suppressor (Bork et al., 2020; Kaleagasioglu et al., 2020; Tamaddoni et al., 2020). The other regulatory pathway is regarded as an inductive pathway, which depends on the formation of Class III PI3K-Beclin-1 complex (Tong et al., 2015; Ohashi et al., 2019).

In the recent few years, the role of autophagy in diseases have been widely explored, including cardiovascular diseases such as myocardial infarction (Liu et al., 2020; Shi et al., 2020) and atherosclerosis (Wang et al., 2019; Cao et al., 2020), neuro-degenerative disorders like multiple sclerosis (Shao et al., 2017; Andhavarapu et al., 2019), metabolic diseases including diabetes (Marasco and Linnemann, 2018; Ding et al., 2020) and obesity (Bartelt and Widenmaier, 2020) and inflammatory and immune-related disorders such as IBD (Dalmasso et al., 2019; Tan et al., 2019; Sola Tapias et al., 2020), and arthritis (Vomero et al., 2018). Although the inflammatory and immune suppressive effect of autophagy has been revealed, some researchers also identified autophagy as a form of cellular death. They pointed out that the induction of autophagy in certain conditions might lead to the so-called “autophagic cellular death” (Denton and Kumar, 2019; Ma et al., 2020; Zhang et al., 2020a). Given those evidence, the mechanisms of autophagy in diseases are complicated. In the following contents, the role of autophagy in IBD will be further described and discussed in detail.

### Autophagy in the Pathogenesis and Progression of IBD

**Figure 1:**
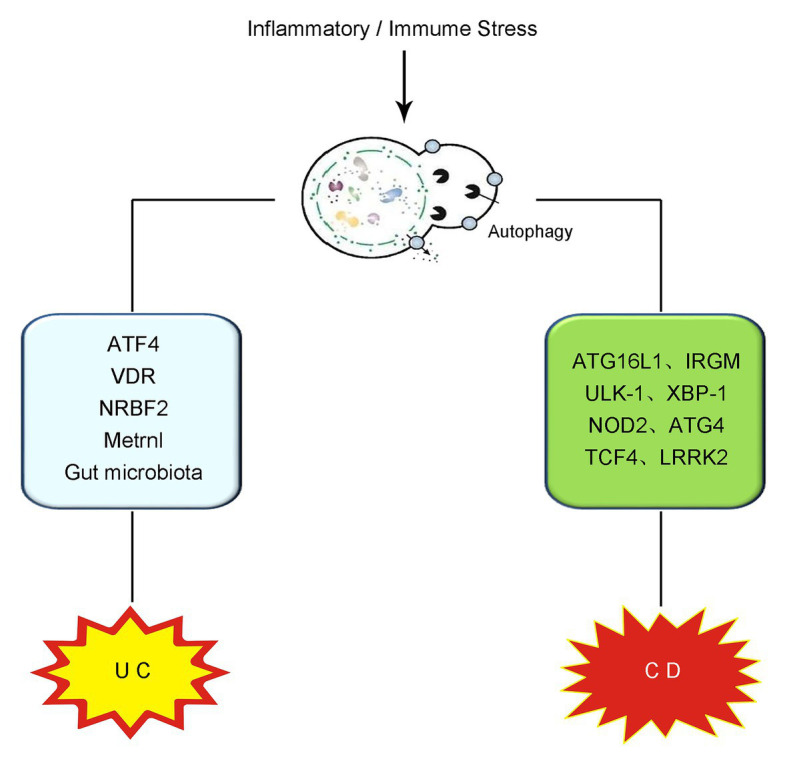
Schematic illustration of the role of autophagy in IBD. The overwhelming inflammatory and immune stress in the gut contributes to the pathogenesis and progression of IBD. On the occurrence of UC, intestinal autophagy is induced and produces effects on UC *via* the modulation of ATF4, VDR, NRBF2, Metrnl, gut microbiota regulation, and so on. In CD, the mutation or deletion of some autophagy-related genes, including ATG16L1, IRGM, ULK-1, XBP-1, NOD2, ATG4, TCF4, and LRRK2, have been reported to be vital in the onset and development of CD. IBD, inflammatory bowel disease; UC, ulcerative colitis; CD, Crohn’s disease; ATF4, activating transcription factor 4; VDR, vitamin D receptor; NRBF2, nuclear receptor binding factor 2; ATG16L1, autophagy-related protein 16-like protein 1; IRGM, immunity-related GTPase M; ULK-1, Unc-51-like kinase-1; XBP-1, X box-binding protein 1 spliced-1; NOD2, nucleotide binding oligomerization domain containing protein 2; TCF4, transcription factor 4; LRRK2, leucine-rich repeat serine/threonine-protein kinase 2.

IBD is recognized as a chronic disorder with pathogenic factors including intestinal microbiota disturbance, mucus barrier damage, gene mutation, and some environmental stimulation (Kelsen and Sullivan, 2017). The incidence of IBD is significantly increased and the age of pathogenesis is becoming younger and younger in the past few decades (Kelsen et al., 2019). IBD comprises two chronic idiopathic inflammatory diseases, namely ulcerative colitis (UC) and Crohn’s disease (CD) (Sairenji et al., 2017). It has been widely reported that autophagy produces an important effect in the onset and development of both UC and CD. In the following contents of this section, the role of autophagy in UC and CD will be discussed, respectively (illustrated in Figure 1).

### Autophagy in UC

UC is a chronic recurrent disorder, affecting all length of the colon and rectum (Ko et al., 2019). So far, it has been revealed that the abnormality of immune system, environment, gut microbiota disturbance, exogenous infection, and certain gene mutations contribute to UC (Feuerstein et al., 2019; Singh et al., 2019; Murphy et al., 2020). However, the specific pathogenesis remains unclarified. UC often spreads from the rectum up to the distal colon and finally involves the inflammatory damage of all the large intestine (Ko et al., 2019; Pravda, 2019). The onset and development of UC often lead to malnutrition syndrome, toxic megacolon, and even colorectal cancer (Burke et al., 1997; Gallinger et al., 2017). In the recent few years, the role of autophagy in UC has been widely explored. In patients with active UC, the level of activating transcription factor 4 (ATF4), an important autophagy-related protein, was significantly decreased in the inflamed intestinal mucosa compared with that in normal mucosa. The findings revealed by that study indicate the reduction of autophagy process on the occurrence of UC (Hu et al., 2019). Recent evidence demonstrated that activating intestinal nuclear receptor vitamin D receptor (VDR) could downregulate intestinal inflammation *via* inducing autophagy-mediated inflammasome suppression (Bakke and Sun, 2018; Karimi et al., 2019; Law et al., 2019). A low VDR expression and dysfunction of vitamin D/VDR signaling was also observed in UC patients (Bakke and Sun, 2018). In addition, Zhou et al. (2018) revealed the mTOR-dependent autophagy flux deficiency in human intestinal epithelial cells from active UC patients. Those findings suggest the correlation between baseline autophagy impairment and the occurrence of UC. However, under the condition of intestinal inflammation, some kinds of microbiota, such as adherent-invasive *Escherichia coli*, was shown to adhere to intestinal epithelial cells and escape autophagy clearance *via* swallowed inside macrophages (Palmela et al., 2018).

In the current studies on UC, dextran sulfate sodium (DSS) is widely applied for the creation of UC animal model, which significantly facilitates the study of UC (Martin et al., 2016; Nunes et al., 2018). In DSS-induced UC models, Wu et al. (2020) reported that nuclear receptor binding factor 2 (NRBF2), a regulatory subunit of the autophagy-related Beclin-1-ATG14-VPS15-VSP34 complex, was necessary for the clearance of apoptotic cells and inflammation suppression during DSS-induced colitis. Studies demonstrated that autophagy contributed to the maintaining and restoration of gut microbiota homeostasis in DSS-induced UC models. Those studies indicate that enhancing the level of autophagy may probably serve as a potential regulator of gut microbiota (Ng, 2015; Xuan et al., 2020; Zha et al., 2020). In addition, it was lately demonstrated by Zhang et al. (2020b) that specific knocking out intestinal Metrnl in epithelial cells produced a detrimental effect UC severity *via* the reduction of autophagy in epithelial cells in DSS-induced colitis models. The AMPK-mTOR-p70S6K signaling, a classic autophagic pathway, was shown to be involved in this process (Zhang et al., 2020b). In consistent with those findings, recent studies conducted by our lab demonstrated that inducing AMPK-mTOR-p70S6K-mediated autophagy through the activation of certain receptors contributed to the attenuation of UC symptoms and suppression of intestinal inflammation in DSS-induced mice models (Ke et al., 2016; Shao et al., 2019a). All of those studies suggest the alleviative role of autophagy in the onset and development of UC.

However, as we have mentioned above, the induction of autophagy under certain conditions or overwhelming induction of autophagy might produce negative effects on cells *via* the triggering of autophagic cellular death. In IBD, it has been reported that deficiency of erbin, a protein required for the polarity of epithelial cells, significantly exacerbated the induction of autophagy process and autophagic cell death in DSS-induced mice models (Shen et al., 2018). The damage of intestinal epithelial cells triggered intestinal inflammation and exacerbated the severity of UC symptoms (Shen et al., 2018). Based on those evidence, to safely taking advantage of autophagy in the treatment of UC, further studies are demanded to explore the pathway to properly induce autophagy.

### Autophagy in CD

CD is a sort of localized or regional enteritis of unknown etiology associated with pathogenetic factors including impaired immunity, environmental stress, familial inheritance, and certain gene mutations (Harb, 2015; Torres et al., 2017; Kienle, 2018). CD often onsets in the region of perineal blind and spreads to the whole length of the colon and distal ileum in combination of some extraintestinal complications (Kienle, 2018). Compared with UC, CD tends to be more strongly affected by genetic factors. It was previously reported by a European study of twins that the proband concordance in monozygotic twins was 58.3 and 6.3% in CD and UC, respectively (Tysk et al., 1988). However, in dizygotic twins, the proband concordance was reduced to 3.9% in CD and no significance in UC (Tysk et al., 1988). According to several genome-wide association study (GWAS) studies conducted in 2007, the mutation of some autophagy-related genes, including *ATG16L1* and immunity-related GTPase M (*IRGM*), two important autophagy-related proteins, were highly related to the pathogenesis of CD (Tysk et al., 1988; Hampe et al., 2007). Buisson et al. (Rioux et al., 2007) revealed that the polymorphisms of autophagy-related genes, including *IRGM*, Unc-51-like kinase-1 (*ULK-1*) and X box-binding protein 1 spliced-1 (*XBP-1*), were associated with macrophage inability to control the replication of CD-associated adherent-invasive *E. coli*. They demonstrated that compared with that from UC patients or healthy controls, the survival rate of adherent-invasive *E. coli* was significantly increased in CD patients with CD-associated polymorphism *IRGM* (*p* = 0.05) and decreased in those with polymorphisms CD-associated *XBP-1* (*p* = 0.026) and CD-associated *ULK-1* (*p* = 0.033; Rioux et al., 2007). In addition, some single nucleotide polymorphisms (SNPs) of *ATG16L1*, such as rs2241880 and rs6754677, have been detected in CD patients and shown to be associated with CD onset (Baradaran Ghavami et al., 2019; Buisson et al., 2019; Kee et al., 2020; Tsianos et al., 2020; Varma et al., 2020). NOD2 is an important autophagy inducer *via* recruiting ATG16L1 into the cell membrane (Park and Jeen, 2019). In CD patients, nucleotide binding oligomerization domain containing protein 2 (*NOD2*) mutation has been shown to lead to the intestinal microbiota homeostasis and over-induction of gut inflammatory responses (Frade-Proud’Hon-Clerc et al., 2019; Waldschmitt et al., 2019; Belyayev et al., 2020). Besides *NOD2*, *IRGM*, *ULK-1*, and *XBP-1*, it was recently reviewed by us that the mutation or deletion of some other autophagy-related genes, including *ATG4*, transcription factor 4 (*TCF4*), leucine-rich repeat serine/threonine-protein kinase 2 (*LRRK2*), and *ATG5* in Paneth cells, were related to the pathogenesis of IBD, especially CD (Wang et al., 2018c). Apart from autophagy-gene mutation, Lu et al. (2014) also showed that microRNA106B and microRNA93 could impair the removal of CD-associated bacteria from epithelial cells though the combination of ATG16L1, thus inhibiting the formation of autophagosomes and autophagy-dependent eradication of intracellular bacteria.

Intrarectal administration of 2,4,6-trinitrobenzenesulfonic acid (TNBS) dissolved in ethanol was initially applied by Morris et al. (1989) for the creation of CD animal models. So far, in TNBS-induced CD models, it was reported that the administration of 3-methyladenine (3-MA) for the blockade of autophagy aggravated the CD symptoms and intestinal inflammation, suggesting the protective effect of autophagy on CD (Macias-Ceja et al., 2017). In addition, Gerster et al. (2015) revealed that high-density lipoprotein (HDL) cholesterol-induced autophagy led to the recruitment of phosphorylated I*κ*B kinase to the autophagosome compartment. Such recruitment prevented nuclear factor-κB (NF-κB)-mediated intestinal inflammation in TNBS-induced CD models. However, it has been demonstrated by Banerjee et al. (2019) that the administration of TNBS *per se* might induce intestinal autophagy toward autophagic cell death, indicating the limitation of TNBS-induced CD models in the study of autophagy.

## Part II: Application of Autophagy Regulators in the Treatment of IBD

As discussed above, autophagy is highly involved in the pathogenesis and progression of IBD. So far, some autophagy regulators are available to us in the treatment of IBD, although most of them have not been applied in the clinical practice. Hereafter, the most popular kinds of such agents, including the well-studied inflammasome inhibitors and intestinal microbiota regulators, will be introduced in the following contents (listed in Table 1).

**Table 1:** Potential pharmacological mechanisms of autophagy regulators in IBD treatment.

Autophagy regulators		Potential pharmacological mechanisms	Targeted autophagy pathway	References
Inflammasome inhibitors taking advantage of autophagy	GL-V9	Inducing AMPK signaling	AMPK signaling	Zhao et al., 2017
	Ginsenoside Rd	Triggering p62-driven mitophagy	AMPK/ULK-1 signaling	Liu et al., 2018
	Palmatine	Inducing mitophagy	PINK1/Parkin-mediated signaling	Mai et al., 2019
	Metformin/MCC950	Modulating HSP90/NLRP3 interaction	AMPK signaling	Saber and El-Kader, 2020
	α7nAChR agonist	Inducing AMPK-mTOR-p70S6K signaling pathway	AMPK-mTOR-p70S6K signaling	Shao et al., 2019a
	CB2R agonist	Inducing AMPK-mTOR-p70S6K signaling pathway	AMPK-mTOR-p70S6K signaling	Ke et al., 2016
Intestinal microbiota regulators taking advantage of autophagy	Vitamin D	VDR-mediated signaling	Acting on ATG16L1	Sun, 2016; Bakke and Sun, 2018
	TREM-1	Restoration of impaired autophagy activity	Unfolded protein response pathway	Kokten et al., 2018
	Galangin	Modulation of inflammatory reaction and myeloperoxidasactivities	AMPK signaling	Xuan et al., 2020

GL-V9, AMP-activated protein kinase activator; AMPK, AMP-activated protein kinase; PINK1, PTEN-induced putative kinase 1; HSP90, heat shock protein 90; NLRP3, NLR family pyrin domain containing 3; α7nAChR, alpha7 nicotinic acetylcholine receptor; mTOR, mammalian target of rapamycin; CB2R, cannabinoid receptor 2; VDR, vitamin D receptor; TREM-1, triggering receptor expressed on myeloid cells-1; ULK-1, Unc-51-like kinase-1; ATG16L1, autophagy-related protein 16-like protein 1.

### Inflammasome Inhibitors Taking Advantage of Autophagy

Inflammasome is recognized as a multi-protein oligomer responsible for activating inflammatory responses (Mariathasan et al., 2004). It belongs to the family of the innate immunity, mainly existing in epithelial cells and most inflammatory and immune cells such as macrophages and dendritic cells in the gut (Lei-Leston et al., 2017; Song and Li, 2018; Shao et al., 2019b). The crosstalk between the inflammasome and autophagy has been well-studied in many disorders (Harris et al., 2017; Deretic and Levine, 2018). So far, several members of inflammasomes have been described, including NLR family pyrin domain containing 1 (NLRP1), NLRP2, NLRP3, NLR family caspase recruitment domain-containing protein 4 (NLRC4), and double-stranded DNA sensors absent in melanoma 2 (AIM2) (Suarez and Buelvas, 2015). Among them, the NLRP3 inflammasome is the most characterized and studied form of inflammasome, which belongs to a member of innate immunity and special form of inflammatory reaction (Huang et al., 2020; Zewinger et al., 2020). The NLRP3 inflammasome comprises three components, including NLRP3 protein, adapter protein apoptosis associated speck-like protein (ASC), and procaspase-1 (Coll et al., 2015). It is mainly produced in inflammatory and immune cells, such as macrophages, NK cells, and lymphocytes (Rathinam et al., 2019). It has been demonstrated that in the recognition of some pathogen-associated molecular patterns (PAMPs) and danger-associated molecular patterns (DAMPs), the reactive components of the NLRP3 inflammasome is accumulated, followed by the formation of the NLRP3 inflammasome complex. The successful formation of the NLRP3 inflammasome induces the transformation of the procaspase-1 to enzymatic caspase-1, which catalyzes the maturation of interleukin (IL)-1β and IL-18 outside cells to stimulate inflammatory reaction cascade (Schroder et al., 2010; Shao et al., 2018; Swanson et al., 2019). So far, it has been revealed by us and other researchers that the over-activation of the NLRP3 inflammasome contributes greatly to the onset and development of IBD (Mangan et al., 2018; Mao et al., 2018; Shao et al., 2019b). According to recent studies, some autophagy regulators have been reported to alleviate IBD *via* suppressing the NLRP3 inflammasome activation (Ke et al., 2016; Zhao et al., 2017; Liu et al., 2018; Mai et al., 2019; Shao et al., 2019a; Saber and El-Kader, 2020).

For instance, it was demonstrated that a small-molecular AMPK activator (GL-V9) significantly degraded the NLRP3 inflammasome complex in macrophages *via* the induction of autophagy, thus protecting against colitis (Zhao et al., 2017). Liu et al. (2018) revealed that Ginsenoside Rd (Rd), a well-known tetracyclic triterpenoid derivative, significantly attenuated the severity of colitis in DSS-induced UC models through the induction of p62-driven mitophagy-mediated NLRP3 inflammasome inactivation. Another natural derivative, Palmatine, was also shown to ameliorate DSS-induced colitis through promoting mitophagy-mediated NLRP3 inflammasome suppression (Mai et al., 2019). In addition, Saber and El-Kader (2020) demonstrated that the combined therapy of metformin and MCC950 produced a protective effect on UC and might become a candidate in the future treatment of UC. They showed that metformin/MCC950 attenuated DSS-induced colitis *via* autophagy-mediated NLRP3 inflammasome inhibition by modulating heat shock protein 90 (HSP90)/NLRP3 interaction. Recently, two studies from us reported the anti-colitis effect of the NLRP3 inflammasome inhibitors taking advantage of autophagy regulation (Ke et al., 2016; Shao et al., 2019a). In one study, we uncovered that the triggering of cholinergic anti-inflammatory pathway by the administration of alpha7 nicotinic acetylcholine receptor (α7nAChR) agonist alleviated DSS-induced colitis. The alleviative process was through autophagy-mediated suppression of the NLRP3 inflammasome-related IL-1β and IL-18 production related to the AMPK-mTOR-p70S6K signaling pathway (Shao et al., 2019a). Similar results were found in another study conducted by us, revealing that cannabinoid receptor 2 (CB2R) agonist contributed to the amelioration of DSS-induced colitis through the induction of AMPK-mTOR-p70S6K-mediated autophagy and inhibition of the NLRP3 inflammasome (Ke et al., 2016).

### Intestinal Microbiota Regulators Taking Advantage of Autophagy

Intestinal microbiota is recognized as diverse bacterial colonies planting in the gut. Intestinal microbiota is generally divided into four phyla, namely Bacteroidetes, Firmicutes, Actinobacteria, and Proteobacteria, among which Bacteroidetes and Firmicutes have been shown to be dominant communities in the gut (Frank et al., 2007; Sender et al., 2016; Zhang et al., 2017). It is widely explored that the disturbance of intestinal microbiota homeostasis is highly involved in the onset and development of IBD (Caruso et al., 2020; Lavelle and Sokol, 2020; Neurath, 2020). As a result, restoring the homeostasis of intestinal microbiota provides a potential and effective therapeutic strategy in the treatment of IBD.

Autophagy has been revealed to play an important role in the regulation of intestinal microbiota. For instance, it was shown by Chu et al. (2016) that the mutation of autophagy-related *ATG16L1* and *NOD2* led to the inactivation of noncanonical autophagy process, resulting in the dysbiosis of gut microbiota in IBD. There is another study on IBD uncovering the effect of autophagy-related gene mutation on intestinal microbiota involved in the induction of the unfolded protein response (UPR; Hooper et al., 2019). In addition, Bel and Hooper (2018) reported the regulatory role of secretory autophagy from Paneth cells in intestinal microbiota in CD patients. They explored the effect of autophagy-related genes on the intestinal defense system. Recently, one of the selective forms of autophagy, named xenophagy, has been largely reported to contribute to modulating gut microbiota in IBD. Xenophagy is recognized as the process of autophagy engulfing intracellular pathogens, because it leads to the elimination of foreign microbes (Sui et al., 2017). It was reported that intestinal epithelial cells with a Crohn’s disease-susceptibility mutation in *ATG16L1* exhibited less xenophagy, thus leading to the epithelial barrier dysfunction (Lopes et al., 2018). In addition, Kim et al. (2019) have demonstrated the involvement of some autophagy-related genes including *ATG16L1*, *IRGM*, ATG7, and p62 in the maintaining of intestinal homeostasis through the triggering of xenophagy. Those studies indicated that taking advantage of autophagy, especially xenophagy, could facilitate the development of new therapeutic opportunities for IBD.

So far, several therapies are available in the alleviation of IBD taking advantage of autophagy-mediated modulation of intestinal microbiota (Sun, 2016; Bakke and Sun, 2018; Kokten et al., 2018; Xuan et al., 2020). It has been reported that vitamin D promoted intestinal autophagy, thus alleviating IBD (Sun, 2016). The signaling of vitamin D/VDR was shown to be beneficial in the maintaining and restoration of intestinal microbiota homeostasis (Bakke and Sun, 2018). In addition, Kokten et al. (2018) demonstrated that the inhibition of triggering receptor expressed on myeloid cells-1 (TREM-1) contributed to the restoration of impaired autophagy activity, thus positively regulating the intestinal microbiota in colitis mice. Xuan et al. (2020) uncovered that Galangin, a natural flavonoid, could fight against DSS-induced colitis through the promotion of autophagy-mediated regulation of gut microbiota. The modulation of inflammatory reaction and myeloperoxidase activities was shown to be involved in this process.

## Conclusion

All in all, recent studies have revealed the role of autophagy in the pathogenesis and progression of IBD. In addition, we are lucky to have several autophagy-related therapies, which have been proven to be effective in the amelioration of IBD. As we have discussed above, several agents taking advantage of autophagy have been demonstrated to be effective in the treatment of IBD. Those agents include some inflammasome inhibitors, especially those targeting on the inflammasome, and some intestinal microbiota regulators. However, although the mechanisms of IBD has been widely investigated, the specific molecular mechanisms of IBD remain unclarified, which largely limits the development of novel treatment for IBD. In addition, because of the complicated effect of autophagy on IBD, to ultimately apply therapeutic strategies taking advantage of autophagy in the clinical practice, further studies are demanded on this issue.

## Author Contributions

B-ZS and YY retrieved concerned literatures and wrote the manuscript. J-SZ and J-HZ designed the table and figure. J-PL and KW revised the manuscript. All authors contributed to the article and approved the submitted version.

### Conflict of Interest

The authors declare that the research was conducted in the absence of any commercial or financial relationships that could be construed as a potential conflict of interest.
